# A Case of Fetal Cleft Lip and Palate Diagnosed by Three-Dimensional Ultrasound During Pregnancy

**DOI:** 10.7759/cureus.79323

**Published:** 2025-02-19

**Authors:** Haruko Fujito, Kazuharu Tanaka, Kensaku Nakai, Yuichiro Awazu, Mayu Kurokawa, Ayako Taniwaki, Masami Hayashi, Aiko Nagashima, Kayoko Nakagawa, Junko Nishio, Osamu Ishiko

**Affiliations:** 1 Obstetrics and Gynecology, Izumiotsu Women's and Children's Hospital, Osaka, JPN

**Keywords:** cleft lip and palate, fetal diagnosis, pregnancy, three-dimensional ultrasound, tomographic ultrasound imaging

## Abstract

We report a case in which a fetal cleft lip and palate were diagnosed by three-dimensional (3D) ultrasound during pregnancy. The patient was a 26-year-old woman with four pregnancies and three deliveries, and her third child had a cleft lip and palate. She had been undergoing antenatal care at our hospital since seven weeks of gestation, and a fetal cleft lip was suspected by a 3D ultrasound taken at 24 weeks of gestation. Up to this time, a diagnosis had not been made due to poor conditions, such as the direction of the fetus. However, the coronal section at 1 mm intervals centered on the palatal area was scanned using tomographic ultrasound imaging (TUI) in a 3D ultrasound at 34 weeks of gestation. The connection between the nasal and oral cavity, as well as a defect in the alveolar ridge, suggested a cleft lip and palate. The mother went into labor at 36 weeks and one day of pregnancy, and a vaginal delivery was performed on the same day. The baby was a 2,164 g boy with an Apgar score of 8/9 and a cleft lip and palate. 3D ultrasound is a useful tool for the detailed depiction of the facial and oral structures of the fetus and may also allow a detailed evaluation of cleft lip and palate.

## Introduction

Cleft lip and palate represent a congenital disorder that poses significant challenges due to associated cosmetic deformities and functional impairments, such as difficulties with feeding, speech development, and dental alignment, with an incidence of approximately one in 500 live births [1]. Additionally, cleft lip and palate have a significant psychological impact on parents, so it is important to perform prenatal ultrasound diagnosis and provide genetic counseling and treatment explanations. Recent technological advancements in three-dimensional (3D) ultrasonography have greatly enhanced the ability to visualize fetal morphological abnormalities, particularly those that are challenging to detect using conventional two-dimensional (2D) ultrasonography. These developments have provided clinicians with improved diagnostic capabilities, enabling more accurate identification of structural anomalies during pregnancy. Despite these advancements, there remains a paucity of published studies specifically addressing the utility of 3D ultrasound in the prenatal diagnosis of cleft lip and palate. It has been reported in Japan that 3D ultrasound is not considered essential for the diagnosis of cleft lip and palate [2]. In this report, we present a detailed case in which a fetal cleft lip and palate were successfully identified using 3D ultrasound imaging during the antenatal period, highlighting its potential benefits for early and precise diagnosis.

## Case presentation

**Figure 1:**
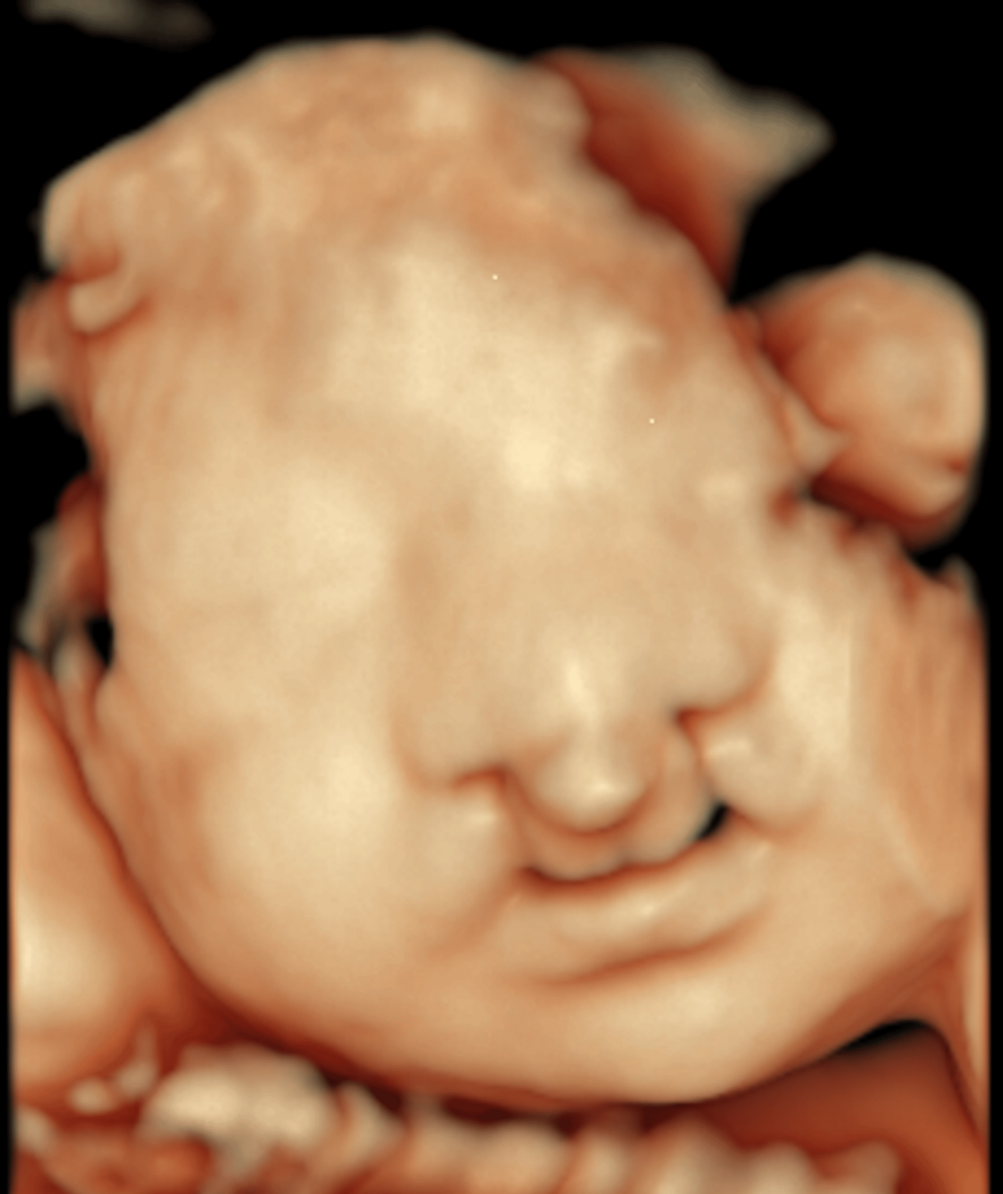
Prenatal detection of bilateral cleft lip using 3D surface rendering ultrasound at 24 weeks of gestation. During ultrasound examination at 24 weeks of gestation, a 3D image was constructed using a surface rendering technique, and a bilateral skin defect was observed on the upper lip, suggesting a cleft lip.

The patient was 26 years old, had four pregnancies and three deliveries (three vaginal deliveries), was 168 cm tall, weighed 58 kg, and had a BMI of 20.5 kg/m2. There were no special notes on her medical history or comorbidities. Her third child had a cleft lip and palate. The pregnancy was spontaneous, and the patient underwent antenatal care at our hospital from seven weeks of gestation. During an ultrasound at 24 weeks of gestation, a GE Voluson E10, eM6C electronic matrix 4D probe (GE HealthCare, Chicago, IL) was used to construct a 3D image using the surface rendering method, and skin defects were observed on both sides of the upper lip, thus suggesting a cleft lip (Figure 1).

**Figure 2:**
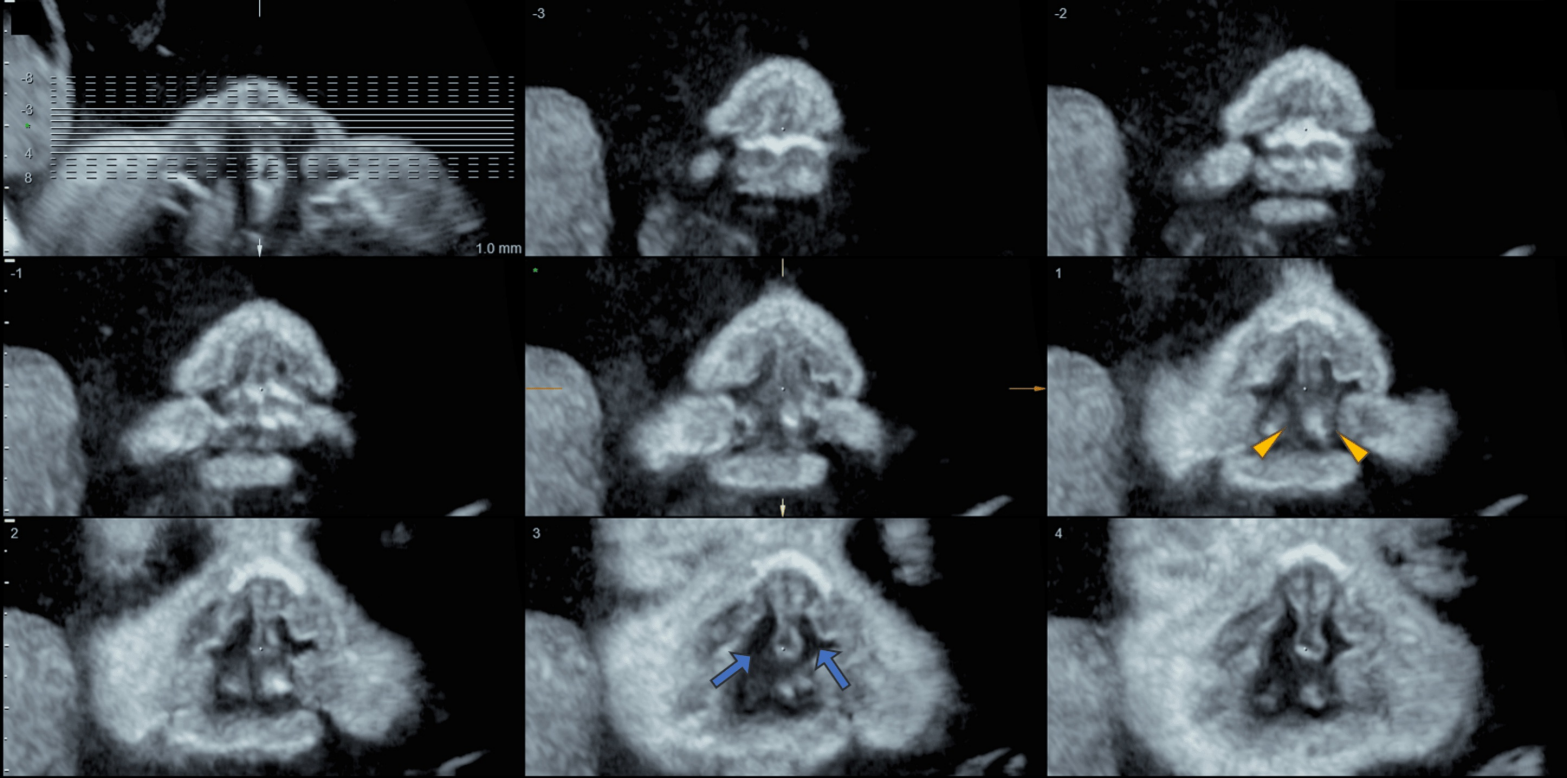
Detection of the cleft jaw using tomographic ultrasound imaging. Tomographic ultrasound imaging was used to scan the coronal section at 1 mm intervals centered on the palatal area, and a cleft jaw was suspected due to the presence of nasal-to-oral cavity traffic (blue arrows) as well as a defect in the alveolar ridge (yellow arrowheads).

Thereafter, the face of the infant could not be confirmed by an ultrasound due to the orientation of the fetus. The coronal section at 1 mm intervals centered on the palatal area was scanned using tomographic ultrasound imaging (TUI) in 3D ultrasound at 34 weeks gestation, and the connection between the nasal and oral cavity, as well as a defect in the alveolar ridge, suggested a cleft lip and palate (Figure 2).

**Figure 3:**
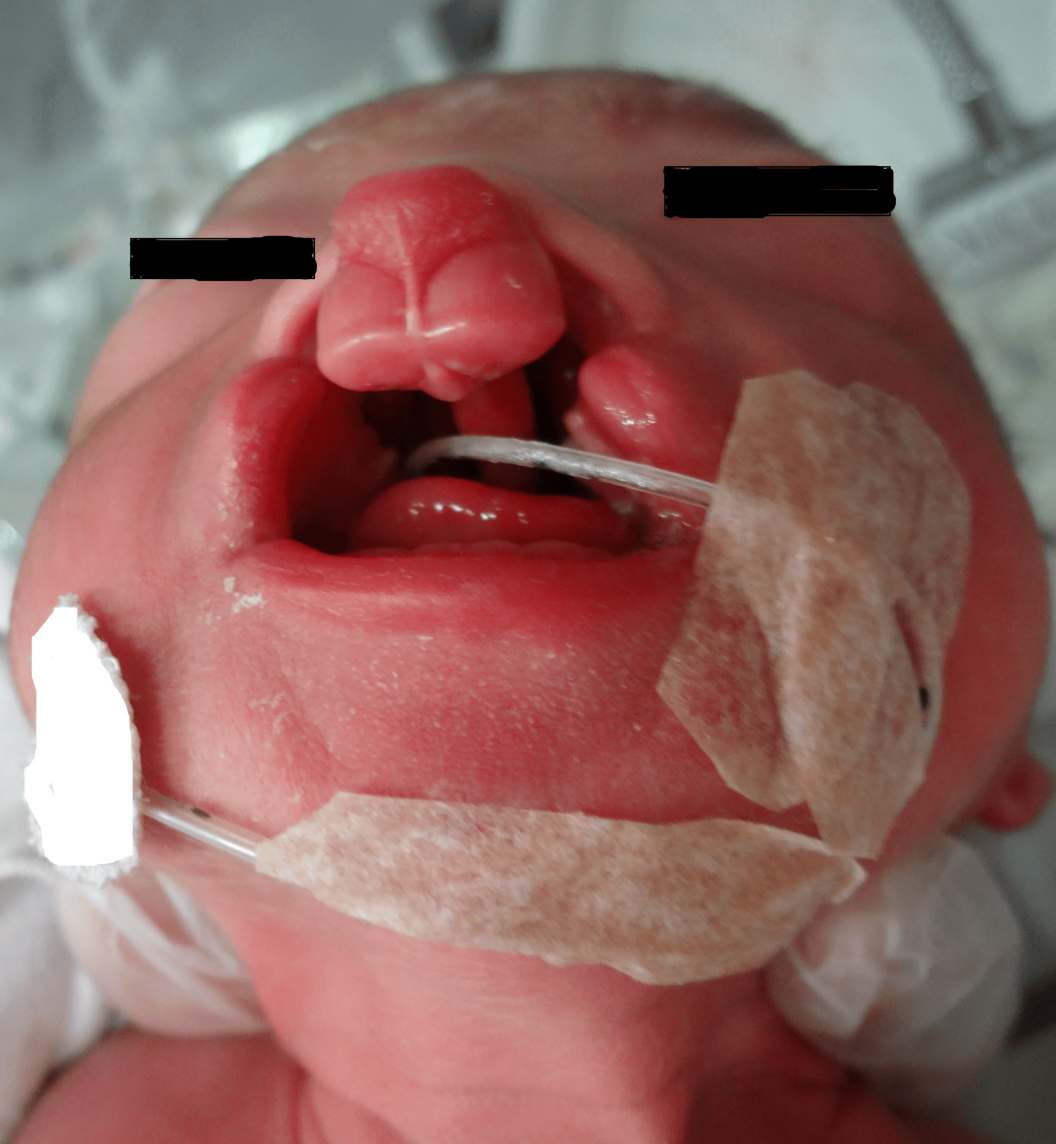
Clinical observations of bilateral cleft lip and palate in the newborn. The hard palate was absent, resulting in a direct connection between the nasal and oral cavities. Additionally, the alveolar ridge was separated on both sides in the anterior portion of the maxilla, indicating bilateral cleft lip and palate.

Genetic counseling was conducted at 34 weeks of gestation in the presence of the attending obstetrician and midwife. It was explained that the fetus had a cleft lip and palate and that there was a possibility that the child would have feeding problems. Other morphological and chromosomal abnormalities that might complicate the condition were also explained. The mother commented that her third child had a cleft lip and palate, so she had a pretty good idea of what to expect. She seemed to accept the situation well. She went into labor at 36 weeks and one day of pregnancy, and a vaginal delivery was performed on the same day. The baby was a 2,164 g boy with an Apgar score of 8/9. The hard palate was absent, resulting in a direct connection between the nasal and oral cavities. Additionally, the alveolar ridge was separated on both sides in the anterior portion of the maxilla, leading to a diagnosis of bilateral cleft lip and palate (Figure 3).

The mother was discharged from the hospital on the 5th day postpartum. The child was fed using a cleft palate nipple with enteral feeding. After consultation with an oral surgeon at another hospital, an artificial palate bed was fabricated to avoid feeding difficulties due to the baby’s poor sucking ability caused by inadequate intraoral negative pressure formation and insufficient nipple pressure. The child was fitted with the artificial palate bed at 13 days of age and was soon feeding well by mouth. The patient was discharged from the hospital on day 26, and her child was treated at the oral surgery department of another hospital.

## Discussion

Cleft lip and palate are a congenital disorder of the face, with or without a combination of cleft lip, cleft palate, or cleft alveolus. The frequency of occurrence is one in 500, with a familial incidence of 8.4% [1]. The cause of its occurrence is unknown, but it is frequently associated with other congenital anomalies. When the child is diagnosed prenatally, the complications should be closely examined [3]. Most cases of cleft lip and palate without congenital disease are considered to be multifactorial genetic disorders rather than chromosomal abnormalities or single gene disorders, and environmental factors such as alcohol consumption and smoking during pregnancy have been reported [4,5]. Problems faced by patients with cleft lip and palate include cosmetic problems due to lip and external nasal deformities, nasal leakage of food and drink, and speech disorders. Dysarthria, exudative otitis media and counter bite, abnormal teeth alignment, and occlusion and oral hygiene in cleft alveolus are common in cases of cleft lip and palate. Postnatal treatment of cleft lip requires an appropriate treatment plan from preoperative treatment, initial plasty, postoperative treatment, and secondary correction [6]. In the case of cleft palate, the use of an artificial palatal bed to close the lanceolate of the hard palate improves feeding, and palatoplasty at nine to 12 months of age is recommended, taking into consideration postoperative language outcome, development of the jaw, and perioperative management [7].

Cleft lip and palate are a morphological abnormality of the face that has a significant psychological impact on the parents. Therefore, prenatal ultrasound diagnosis should be performed, and genetic counseling and postnatal treatment of the child should be explained. Many of the patient's family members feel reassured by the information about the treatment, and the psychological burden caused by the postnatal facial morphology abnormality is reduced, thus leading to a positive attitude toward the treatment. However, the prenatal diagnosis rate of cleft lip and palate in the low-risk group without congenital diseases or chromosomal abnormalities is only 14-58% [8,9], and further improvement of diagnostic accuracy is considered important.

Recently, the development of 3D ultrasonography has made it possible to perform prenatal diagnosis of fetal morphological abnormalities that are difficult to diagnose with conventional 2D ultrasonography. However, few papers in Japan have reported the usefulness of a 3D ultrasound in diagnosing cleft lip and palate. Although a 3D ultrasound is useful in the sense that showing family members 3D images can facilitate the communication of specific treatment plans and, therefore, reduce psychological anxiety, it has been reported that 3D ultrasound is not essential for the diagnosis of cleft lip and palate [9]. However, there are reports from overseas that the multiplanar reconstruction mode of a 3D ultrasound is useful in the diagnosis of cleft lip and palate [10]. The palate is surrounded by hard tissues, thus making it difficult to observe with an ultrasound. Moreover, the prenatal diagnosis of cleft palate is less frequent than that of cleft lip and palate. Kawai et al. reported that cleft lip or cleft palate was diagnosed prenatally in the majority of 75 cases treated at a cleft lip and palate center from 2010 to 2016, and cleft palate alone was not diagnosed prenatally in any case [11]. On the other hand, Ji et al. reported that 3D ultrasonography has improved the diagnostic accuracy of cleft lip and palate, compared to conventional 2D ultrasonography [12].

In this case, with the use of TUI of 3D ultrasound to continuously delineate the coronal section, we were able to observe the section from the upper lip to the palate and to evaluate in detail the presence and extent of the cleft palate. Thus, a 3D ultrasound is a useful tool for the detailed delineation of the facial and oral structures of the fetus, and it is expected to have the potential for detailed evaluation of not only cleft lip and palate but also cleft palate only.

## Conclusions

In this case, we successfully utilized 3D ultrasound to diagnose both cleft lip and palate in a patient with a suspected fetal cleft lip and palate. This advanced imaging technique allowed us to achieve a more precise prenatal diagnosis, enabling us to provide the patient and their family with detailed information about the condition, as well as comprehensive genetic counseling and guidance on potential postnatal management and treatment options.
